# Tunable graphene-based hybrid plasmonic modulators for subwavelength confinement

**DOI:** 10.1038/s41598-017-05172-9

**Published:** 2017-07-12

**Authors:** Sheng Qu, Congcong Ma, Hongxia Liu

**Affiliations:** Xidian University, Key Laboratory for Wide Band Gap Semiconductor Materials and Devices of Education, School of Microelectronics, Xi’an, 710071 China

## Abstract

Electro-optical modulators which work at the near-infrared range are significant for a variety of applications such as communication and sensing. However, currently available approaches result in rather bulky devices which suffer from low integration and can hardly operate at low power consumption levels. Graphene, an emerging advanced material, has been widely utilized due to its tunability by gating which allows one to realize active optical devices. Plasmonic waveguides, one of the most promising candidates for subwavelength optical confinement, provide a way to manipulate light on scales much smaller than the wavelength. In this paper, we combine the advantages of graphene and plasmonic waveguides and propose a tunable graphene-based hybrid plasmonic modulator (GHPM). Considering several parameters of the GHPM, the modulation depth can reach approximately 0.3 dB·μm^−1^ at low gating voltages. Moreover, we combine GHPM with metal-insulator-metal (MIM) structure to propose another symmetrical GHPM with a modulation depth of 0.6 dB·μm^−1^. Our modulators which utilize the light-matter interaction tuned by electro-doped graphene are of great potential for many applications in nanophotonics.

## Introduction

The need for fast, compact and low-energy consumption electro-optical modulators has motivated research into optical structures capable of guiding light with deep subwavelength confinement^1^. High-integration density of optical devices and miniaturization of device size remain major challenges in micro and nanotechnology. Due to the existence of the diffraction limit, traditional optical devices are difficult to operate in micro-nano size. Plasmons, the collective oscillations of valence electrons in conducting materials, possess a number of appealing properties for photonic technologies, the most salient of which are (1) their small spatial extension compared with the light wavelength; (2) their strong interaction with light; (3) the huge optical enhancements produced by the strong interaction. Compared to other waveguides, plasmonic waveguides can break the diffraction limit and provide subwavelength optical confinement by storing optical energy in electron oscillations within dissipative metallic regions^2–8^. Therefore, plasmonic waveguides have been regarded as one of the most promising candidates for manipulating light on nanoscale.

Graphene, a single layer of carbon atoms in a hexagonal lattice, holds an attractive potential to realize fast and compact optoelectronic devices. For applications in optical modulators, graphene has several unique characteristics. (1) Broad optical bandwidth. Graphene has a constant absorption from visible to infrared wavelengths which covers the optical fiber communication bandwidth^9, 10^. (2) High speed operation. With a carrier mobility as high as 200,000 cm^2^/(Vs) at room temperature (Actually, the real mobility of monolayer graphene is much smaller than the theoretical value and we use ~10^4^ cm^2^/(Vs) in our simulation), graphene-based electronics may have the potential to operate at ultrafast condition (over hundreds of GHz)^11^. The Fermi energy and hence the optical absorption can be rapidly modulated via band-filling effect^12, 13^. (3) Strong light-graphene interaction. A monolayer graphene can possess a much higher optical absorption of ~2.3%, which is much higher than that of compound semiconductors at the same thickness^14^. What’s more, the strong plasmonic response of graphene also makes it a promising material to manufacture highly integrated devices. For graphene plamsonics, there are two classes: propagating (waveguide) and localized (nanostructure) graphene plasmons. And for coupling of graphene plasmons, there are also two classes: near-field (directly) and far-field (phase) coupling. Recently, some novel processes based on phase-coupling scheme of localized graphene plasmon resonances have been proposed to realize ultra-high contrast optical modulations^15, 16^. (4) Compatibility with CMOS processing. During the past decade, graphene-based optoelectronics integrated with CMOS processing have been already demonstrated that makes it attractive in numerous applications such as graphene optical modulators^17^, detectors and sensings^18, 19^. Based on these advantages, graphene-based modulators will be competitive with ultrafast modulation speed and low operation voltage across a broad optical bandwidth. Although there are many kinds of graphene-based modulators has been demonstrated^17, 20^, it is still largely unexplored to combine the graphene with plasmonic waveguides.

In this paper, we propose a tunable graphene-based hybrid plasmonic modulator (GHPM) combining the advantages of graphene and plasmonic waveguides. By realistic simulation, a significant modulation depth of ~0.3 dB·μm^−1^ can be obtained at the wavelength of 1550 nm. What’s more, combining with the metal-insulator-metal (MIM) structure, the modulation depth can reach 0.6 dB·μm^−1^. These results open a viable route toward electro-optical modulation within technologically important frequency range.

## Materials

**Figure 1:**
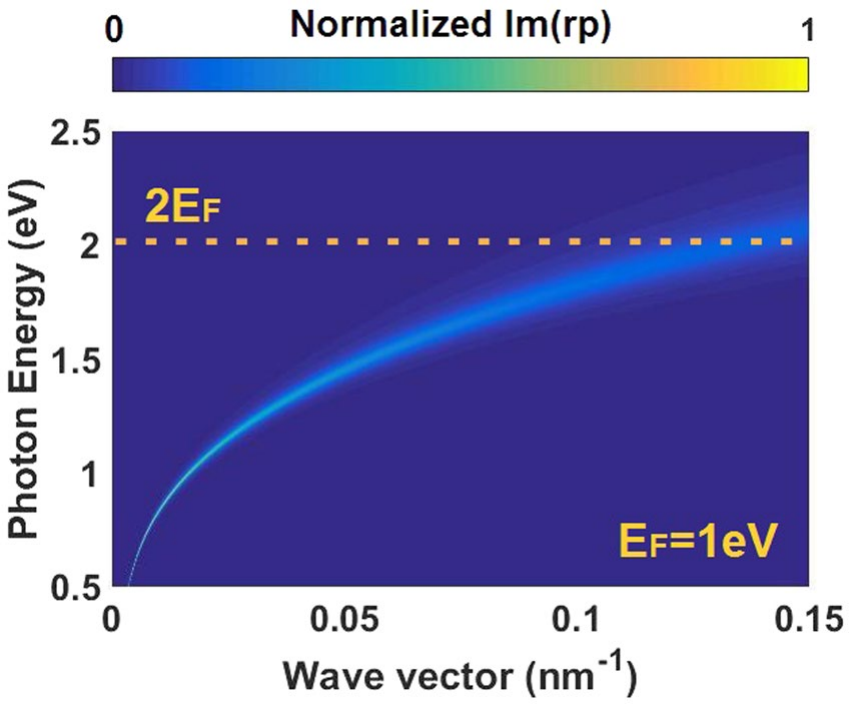
Plasmon dispersion of monolayer graphene when the graphene is doped (For clarify, here we use highly doped graphene with *E*_F_ = 1 eV.), as illustrated by the photon-energy and parallel-wave-vector dependence of the imaginary part of reflection coefficient for p-polarization. Interband absorption produces strong plasmon quenching when the photon energy exceeds 2*E*_F_ (i.e., above the yellow dash line).

Before describing the structure of our device, it is essential to explore the optical response of monolayer graphene firstly. Whether our device is modulated by the exciting graphene plasmons or interband absorption, the result is demonstrated as follows. The graphene plasmons dispersion is shown in Fig. 1. Incidentally, we use random phase approximation (RPA)^21–24^ to simulate the plasmons dispersion.

**Figure 2:**
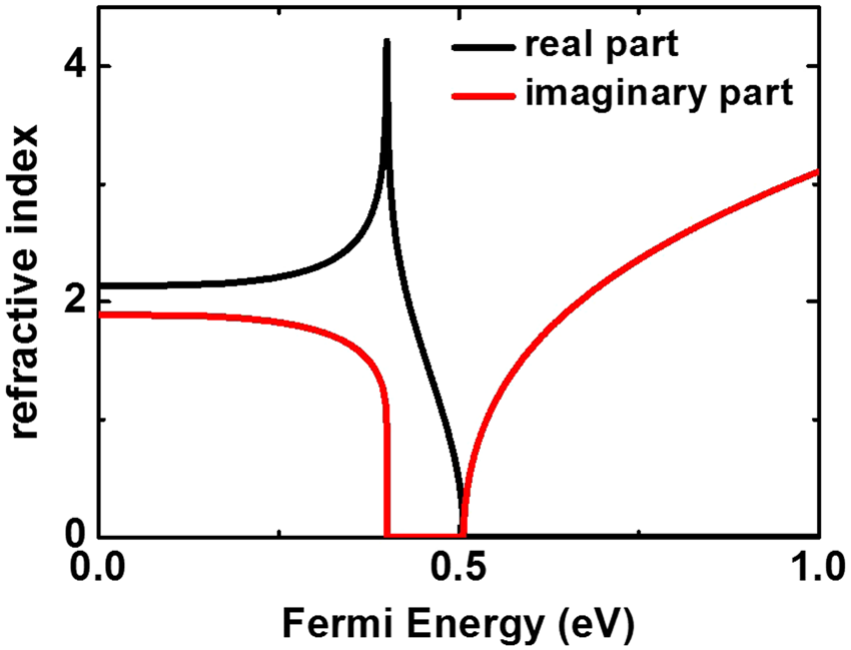
The refractive index of monolayer graphene varies with the Fermi energy (details see Methods). The black line and the red line represent the real part and imaginary part of the refractive index, respectively.

Figure 1 has illustrated that when ћω is close to or greater than 2*E*_F_, the property of graphene performs as the interband absorption due to the strong quantum quenching via coupling to interband absorption. And in our research (i.e., *E*_F_ ranges from 0 to 0.4 eV), ћω is always larger than 2*E*_F_ so that the property of graphene shows as interband absorption instead of plasmon modes excitation. Incidentally, interband absorption modulates the propagating modes by affecting the graphene conductivity derived by Kubo formula (details see Methods) and further changing the refractive index as shown in Fig. 2.

As shown in Fig. 2, one can clearly find the extreme value of imaginary part of refractive index when *E*_F_ ≈ 0.4 eV. Due to the effect of optical Pauli blocking here, the optical absorption of graphene is close to zero. This effect is based on the change of graphene optical conductivity induced by the shift of the Fermi energy produced by electrical doping (details see Methods). To completely block optical absorption in graphene at the wavelength of 1550 nm, we need to shift the Fermi energy from pristine values of *E*_F_ = 0 to *E*_F_ = 0.4 eV.

## Results

### GHPM structure and properties

**Figure 3:**
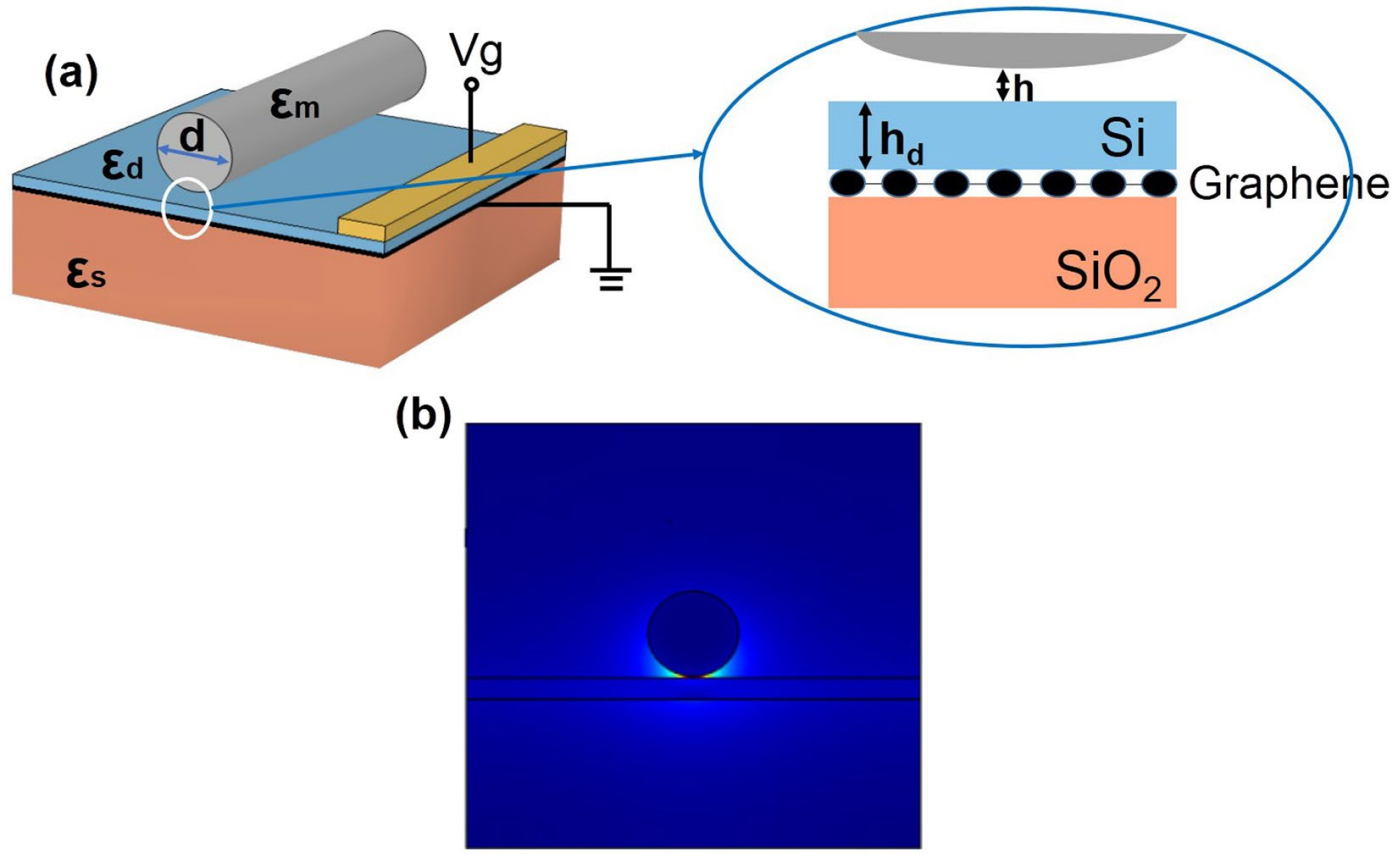
The structure and mode distributions of the designed GHPM. (**a**) Three dimensional structure and cross section of the proposed hybrid modulator. (**b**) The mode distributions of the designed modulator at a 1550 nm wavelength where d = 200 nm, h = 4 nm and h_d_ = 50 nm.

In this paper, we propose a plasmonic modulator tuned by a monolayer graphene. This device is compatible with the conventional integrated circuits process. Figure 3a depicts the three-dimensional layout of the GHPM and the cross-section structures, respectively. The structure consists of a silver cylindrical nanowire of permittivity ε_m_ and diameter d separated from a silicon slab of permittivity ε_d_ and thickness h_d_ by a nanoscale dielectric gap of permittivity ε_s_ and thickness h. And a monolayer graphene is inserted between the silicon slab and the silica substrate. The dielectric material surrounding the silver nanowire is the same material used as the substrate. In our simulation, the electrodes are far enough on the guide mode so that those effects can be neglected. In order to clearly display the structure of our device, we neglect the cladding surrounded the metal nanowire in Fig. 3a. For the practical applications, we can use a grating to couple light into a plasmon-propagating mode which can be tuned by gated graphene via electrical doping (details see Methods). The mode distribution of the proposed modulator is shown in Fig. 3b. One can clearly see that the electric field intensity is tightly confined to the metal surface in front of the silicon slab.

In the simulations, we choose the working wavelength at 1550 nm. Figure 3a illustrates the structure of the designed GHPM, where ε_d_ = 12.25 and ε_s_ = 2.25 at 1550 nm^25, 29^. The refractive index of Ag is 0.14526 + i11.359^26^. At first, the cylinder diameter, gap thickness and silicon slab thickness are set at 200, 4 and 50 nm, respectively. The conductivity of graphene is derived above. In the following study, we study the GHPM firstly, in which the modulation is realized by shifting the Fermi energy by electrical doping from 0 eV to 0.4 eV at the wavelength of 1550 nm. Then, we adjust the cylinder diameter d, the dielectric gap width h and the silicon slab width h_d_ to control the modulation depth.

**Figure 4:**
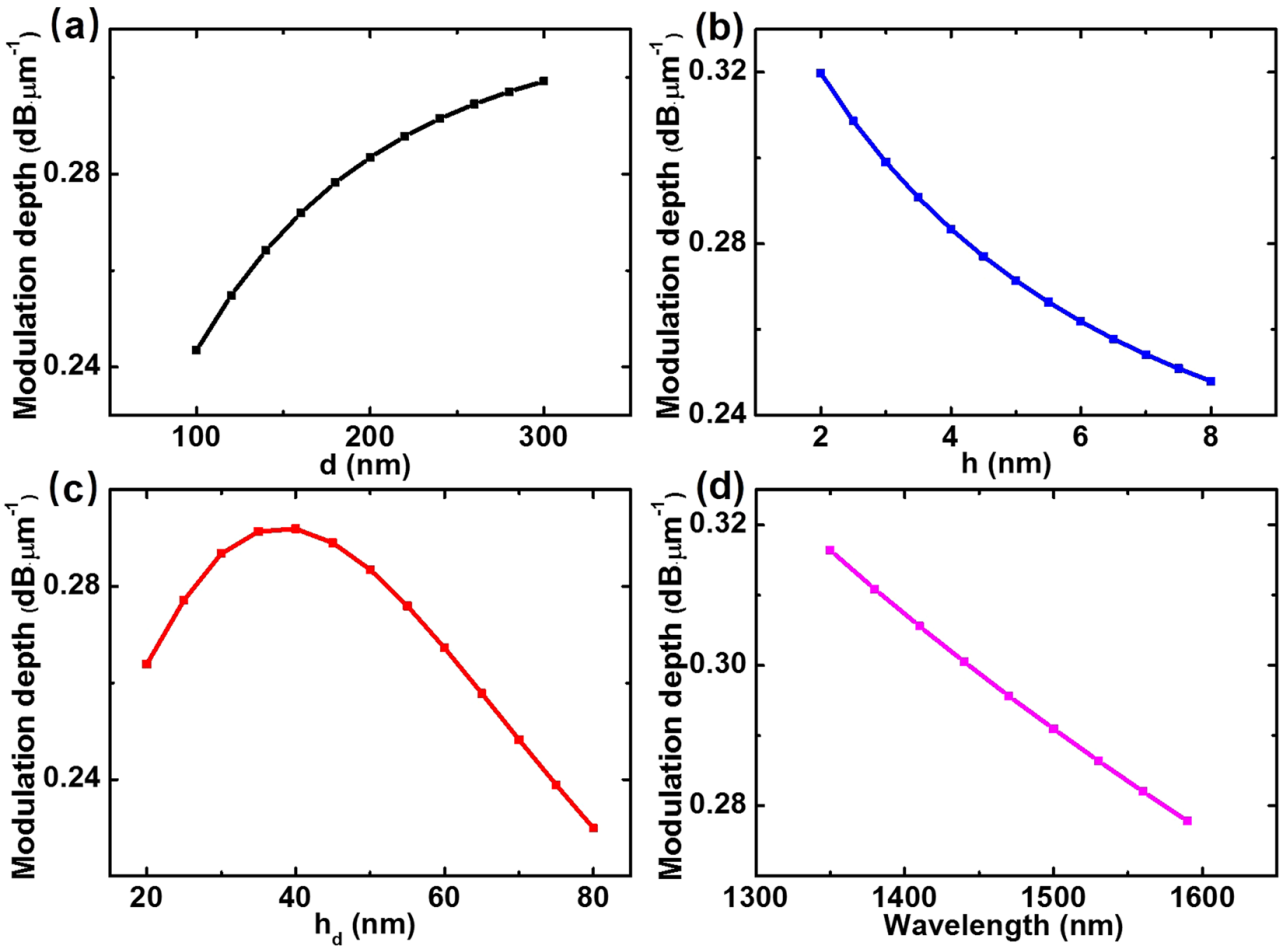
The modulation depth of GHPM varies with the cylinder diameter d, the dielectric gap thickness h and the silicon slab thickness h_d_, respectively. (**a**) The cylinder diameter d varies from 100 to 300 nm where h = 4 nm and h_d_ = 50 nm. (**b**) The gap thickness h varies from 2 to 8 nm where d = 200 nm and h_d_ = 50 nm. (**c**) The silicon slab thickness h_d_ varies from 20 to 80 nm where d = 200 nm and h = 4 nm. (**d**) The modulation process of GHPM is achieved for a broad band of wavelengths from 1350 nm to 1600 nm.

Here we mainly analyze the modulation depth of this proposed modulator. Compared to the previous hybrid modulators (the modulation depth is ~0.03 dB·μm^−1^ ^27^), the modulation depth of our modulator can reach approximately as high as 0.3 dB·μm^−1^ at low gating voltages where the cylinder diameter, gap thickness and silicon slab thickness are set at 200, 4 and 50 nm, respectively. Meanwhile the insertion loss is approximately 0.02 dB·μm^−1^ which can be neglected. To further research the relationship between modulation depth and the structure parameters, we calculate the modulation depth by varying the cylinder diameter d, the dielectric gap thickness h and the silicon slab thickness h_d_. Relevant results are shown in Fig. 4.

As mentioned above, a significant consequence is that the modulation depth is a trade-off between the mode field intensity and the distance of graphene layer to the center of mode field. As shown in Fig. 4a, by simulation we can find that the mode field intensity increases with the diameter, and the distance of graphene layer to the center of mode field is a constant simultaneously. Thus the modulation depth gradually increases with the cylinder diameter. As shown in Fig. 4b, one can clearly find that the modulation depth decreases with the dielectric gap. This can be interpreted that the distance of graphene layer to the center of mode field becomes larger with the increase of the dielectric gap. Meanwhile, the mode field intensity decreases by simulation analysis. The dependence of modulation depth on the silicon slab thickness is shown in Fig. 4c. One can clearly find that the modulation depth first increases and then decreases with the thickness of the silicon slab. With the silicon slab thickness increasing, the mode field intensity gradually increases, however the distance from the graphene layer to the mode center becomes larger. Thus this trade-off leads to the phenomenon shown in Fig. 4c. As shown in Fig. 4d, a modulation depth greater than 0.27 dB·μm^−1^ is achieved for a broad band of wavelengths, from 1350 nm to 1600 nm.

The proposed GHPM combines the advantages of graphene and plasmonic waveguides. We utilize the tunability of graphene and the subwavelength optical confinement of plasmonic waveguides. Considering several parameters of the GHPM, the modulation depth can reach approximately 0.3 dB·μm^−1^ at low gating voltages. In fact, the location of graphene layer relative to the mode is the true nature causing the change of modulation depth with the parameters.

### SGHPM structure and properties

**Figure 5:**
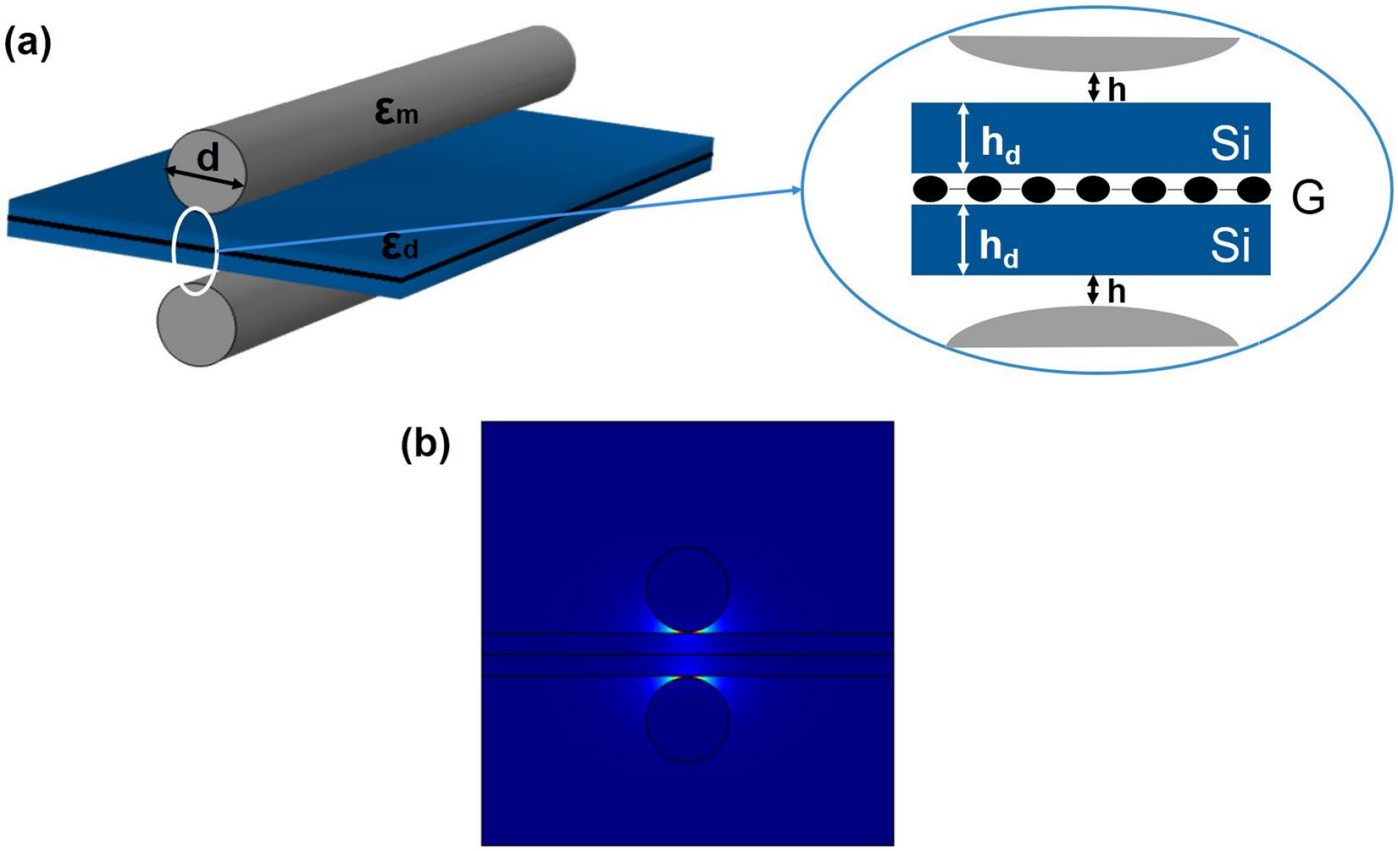
The structure and mode distributions of SGHPM. (**a**) Three dimensional structure of the hybrid modulator. (**b**) The mode distributions of the designed modulator at the wavelength of 1550 nm where d = 200 nm, h = 4 nm and h_d_ = 50 nm.

Based on the structure proposed above, here we design another plasmonic modulator utilizing metal-insulator-metal (MIM) waveguides^28^. As shown in Fig. 5a, this structure consists of two identical metal nanowires symmetrically placed on each side of a thin silicon slab. And a monolayer graphene is inserted in the center of the silicon slab. The material surrounding the two silver nanowires and the electrodes are not displayed in Fig. 5a to lighten the geometry. Due to this structure consists of two symmetrical GHPM, we call it a symmetrical graphene-based hybrid plasmonic modulator (SGHPM).

Actually, there are many proposed plasmonic waveguides utilizing the insulator-metal-insulator (IMI) structure to achieve long range propagation^29^. Although IMI waveguides suffer lower propagation loss, the MIM waveguides provide tighter modes confinement near the graphene layer due to the SPPs modes coupling. As the modes distribution shown in Fig. 5b, one can easily find tighter modes confinement than which is shown in Fig. 3b. Thus this structure can improve the tunability of plasmonic modulators. By numerical analysis, the propagation length (~10 μm) of the modes supported by this MIM waveguide can satisfy the demands of this proposed modulator. The modulation depth of SGHPM can reach approximately 0.6 dB·μm^−1^ which is higher than that of GHPM where the cylinder diameter, gap thickness and silicon slab thickness are set at 200, 4 and 50 nm, respectively. Meanwhile the insertion loss is 0.05 dB·μm^−1^ which is much smaller than the value of modulation depth.

**Figure 6:**
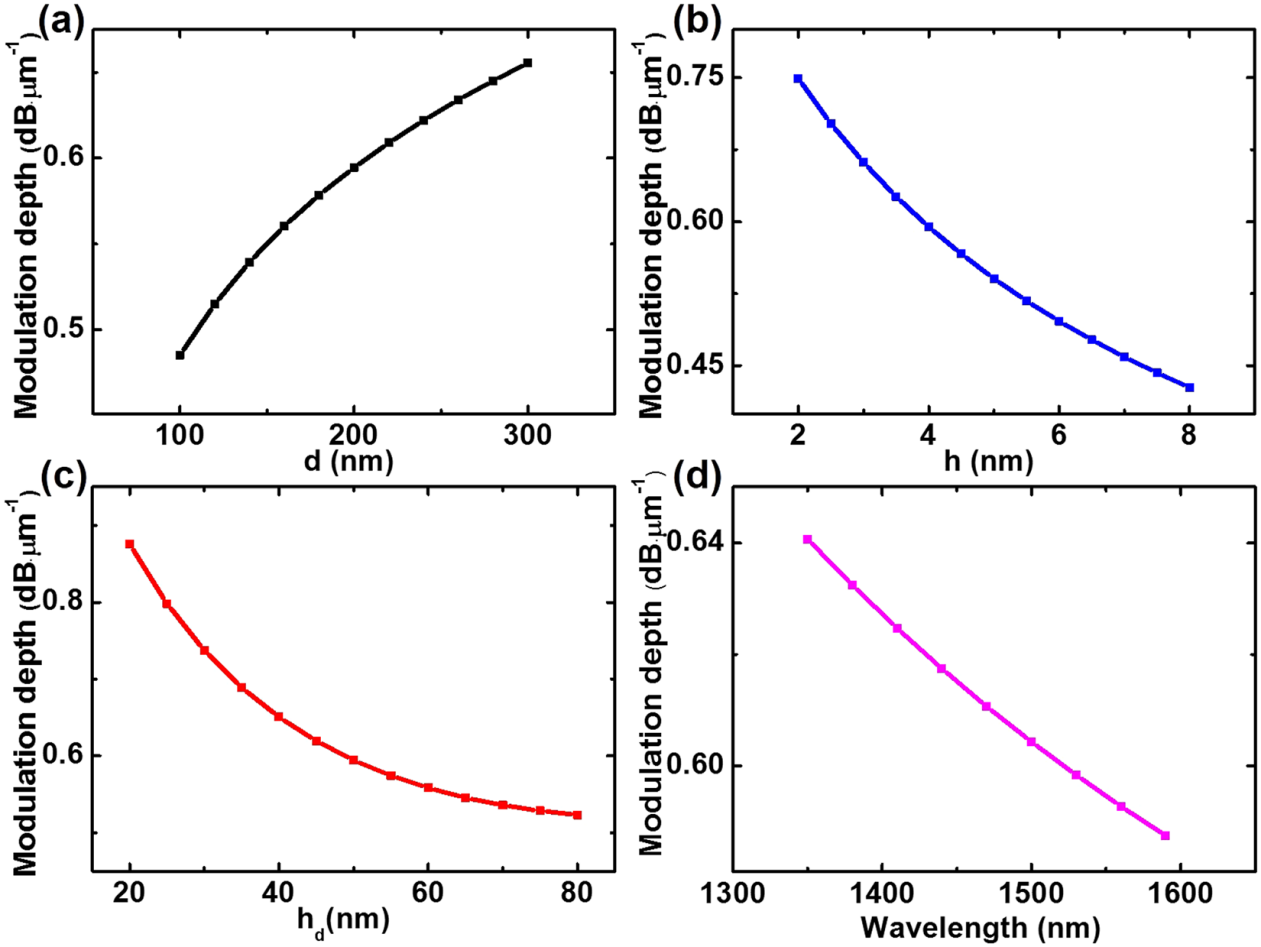
The modulation depth of SGHPM varies with the cylinder diameter d, the dielectric gap thickness h and the silicon slab thickness h_d_, respectively. (**a**) The cylinder diameter d varies from 100 to 300 nm where h = 4 nm and h_d_ = 50 nm. (**b**) The gap thickness h varies from 2 to 8 nm where d = 200 nm and h_d_ = 50 nm. (**c**) The silicon slab thickness h_d_ varies from 20 to 80 nm where d = 200 nm and h = 4 nm. (**d**) The modulation process of SGHPM is achieved for a broad band of wavelengths from 1350 nm to 1600 nm.

**Figure 7:**
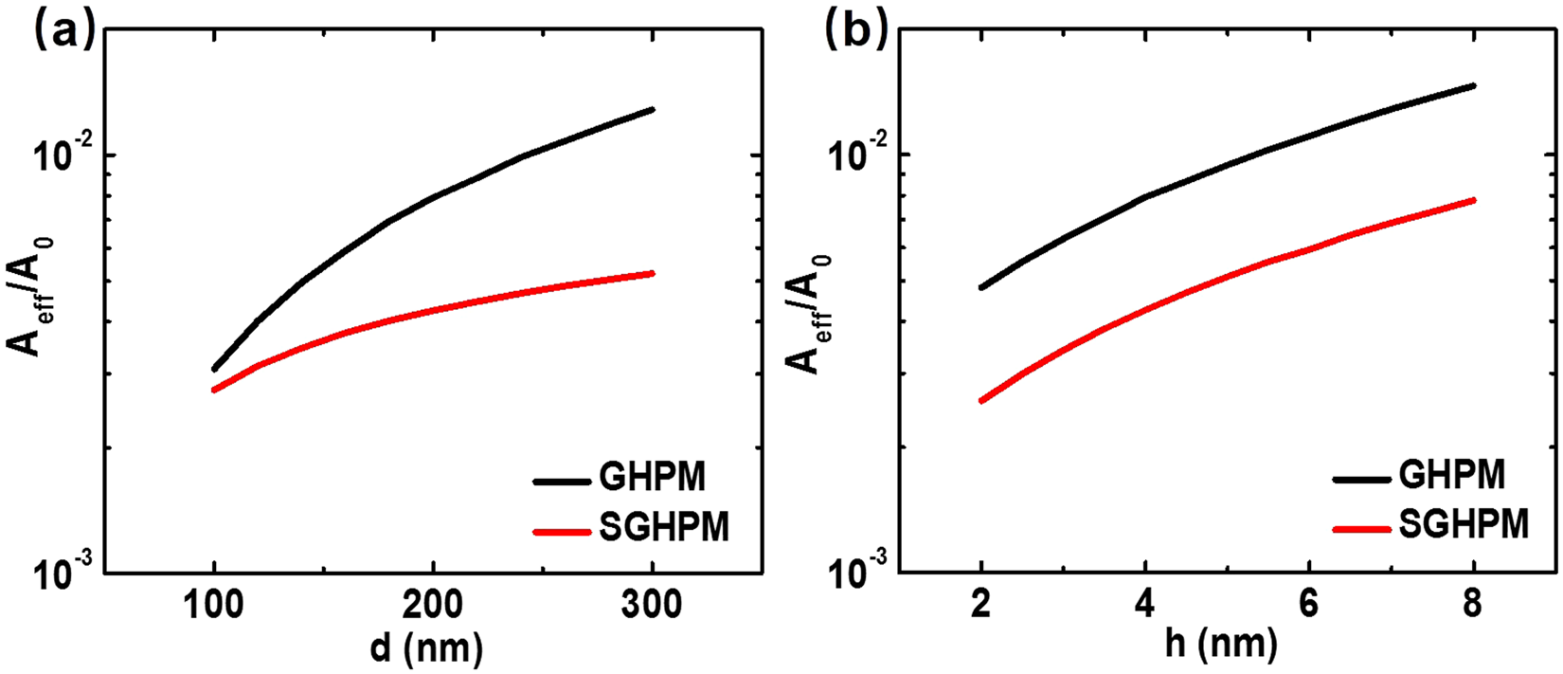
The normalized effective mode area (*A*_*eff*_ */A*_*0*_) varies with the cylinder diameter d and the dielectric gap thickness h for the GHPM (black lines) and SGHPM (red lines), respectively. (**a**) The cylinder diameter d varies from 100 to 300 nm where h = 4 nm and h_d_ = 50 nm. (**b**) The dielectric gap thickness h varies from 2 to 8 nm where d = 200 nm and h_d_ = 50 nm.

As shown in Fig. 6, due to this SGHPM can be considered as two symmetrical GHPM, the dependence of the modulation depth shows similar properties as GHPM. The only difference is the modulation depth decrease with the thickness of the silicon slab. Here we find that the MIM structure can decrease the effective mode area as shown in Fig. 7. Compared SGHPM with GHPM, the MIM structure can make SPP modes more concentrated on the surface of metal nanowires. This result in a decrease of the effect of increase silicon slab thickness on the increase of modes intensity. However, the distance from graphene to mode center increases with the silicon slab which reduces the tunability of the graphene layer and leads to a decrease in the modulation depth as shown in Fig. 6c. As shown in Fig. 6d, a modulation depth greater than 0.58 dB·μm^−1^ is achieved for a broad band of wavelengths, from 1350 nm to 1600 nm. Another significant parameter shown in Fig. 7 is the normalized effective mode area which is used to explain the subwavelength confinement in our modulator.

As shown in Fig. 7, we calculate the normalized effective mode area of the two proposed modulators (details see Methods). One can easily find that both modulators show tight mode subwavelength confinement which ensures that our modulator can achieve subwavelength modulation. Moreover, the normalized effective mode area of both modulators increases with the cylinder diameter and dielectric gap thickness. Combined with conclusions above, we have analyzed the overall performance of our subwavelength plasmonic modulators. Several other parameters (i.e. the modulation speed and energy consumption) are shown in Methods.

## Discussion

In this paper, we have proposed a tunable graphene-based hybrid plasmonic modulator (GHPM) combining the advantages of graphene and plasmonic waveguides. The modulated optical signals are obtained by electrically tuning of the graphene’s refractive index and the subwavelength confinement is achieved via plasmonic waveguides. The numerical simulations at the wavelength of 1550 nm have shown that the GHPM with relatively low loss and nanoscale light confinement can gain an attractive modulation depth of nearly 0.3 dB·μm^−1^. What’s more, the modulation depth of the SGHPM is achieved a higher value (~0.6 dB·μm^−1^) than that of the GHPM. Our hybrid modulators have a great potential in graphene-based nanoscale optical devices.

## Methods

### Properties of monolayer graphene

#### Parameters of monolayer grapheme

In our research, as relative discussion proposed in the text, the optical response of graphene is described by the conductivity which is modeled utilizing Kubo formula^30^. At temperature T = 296 K, the conductivity σ of graphene is given by

1 $${\sigma }_{{\rm{total}}}={\sigma }_{{\rm{intra}}}+{\sigma }_{\mathrm{inter}},$$

2 $${\sigma }_{{\rm{intra}}}={\sigma }_{0}\frac{4{E}_{F}}{\pi \hslash ({\tau }_{1}-i\omega )},$$

3 $$\begin{array}{rcl}{\sigma }_{\mathrm{inter}} & = & {\sigma }_{0}(1+\frac{1}{\pi }\arctan \frac{\hslash \omega -2{E}_{F}}{\hslash {\tau }_{2}}-\frac{1}{\pi }\arctan \frac{\hslash \omega +2{E}_{F}}{\hslash {\tau }_{2}})\\  &  & -i{\sigma }_{0}\frac{1}{2\pi }\,\mathrm{ln}\,\frac{{(2{E}_{F}+\hslash \omega )}^{2}+{(\hslash {\tau }_{2})}^{2}}{{(2{E}_{F}-\hslash \omega )}^{2}+{(\hslash {\tau }_{2})}^{2}}\end{array},$$

where σ_total_ is the conductivity of monolayer graphene which consists of intraband conductivity σ_intra_ and interband conductivity σ_inter_ and *ћ* is the reduced Plank’s constant. σ_0_ = 60.8 μS, λ = 1550 nm, relaxation time τ_1_ = 1.2 ps for interband conductivity and τ_2_ = 10 fs for intraband conductivity are constants, so that the conductivity of graphene is calculated as a function of Fermi level *E*_F_. The permittivity of monolayer graphene is derived by

4 $${\varepsilon }_{{\rm{g}}}=1+i{\sigma }_{total}/\omega {\varepsilon }_{0}{\rm{\Delta }},$$

where Δ is the thickness of graphene assumed to be 0.7 nm in simulation. And the refractive index of graphene is derived by

5 $${n}_{g}={\varepsilon }_{g}^{1/2}.$$

#### Electrical tuning approach

In our proposed modulators, the carrier concentration in monolayer graphene is dynamically controlled by employing a top-gate voltage^31^, as shown in Figs 3a and 5a. The Fermi energy of graphene tuned by applied voltage can be estimated using the parallel capacitor model as

6 $$|{E}_{F}|=\hslash {v}_{f}\sqrt{\pi \alpha |{V}_{g}|},$$

where *E*_F_ is the Fermi level at gate voltage V_g_, v_f_ is the Fermi velocity ~10^6^ m/s, and α = ε_0_ε_d_/(e × h_d_) is the capacitor constant. In the case of h_d_ = 20 nm, when the gate voltage is swept from 0 to ~8 V, *E*_F_ changes from 0 to ~0.4 eV to achieve modulation. Thus the proposed modulators can be electrical tuned through electric field effect.

### Properties of plasmonic modulators

The mode properties are investigated by means of the finite-element method (FEM) with the scattering bound condition.

#### Modulation depth

The modulation depth *Mp* is given by

7 $$Mp=\frac{ER}{L}\approx 70.42\times ({k}_{\max }-{k}_{\min }),$$

where *ER* is the extinction ratio and *L* is the active region length. The *ER* is calculated by

8 $$ER=10\,\mathrm{log}(\frac{{P}_{\max }}{{P}_{\min }})\approx 70.42\times L\times ({k}_{\max }-{k}_{\min }),$$

where *P*_max_ and *P*_min_ is the maximum and minimum electromagnetic power, respectively. The *P*_max_ and *P*_min_ are given by

9 $${P}_{\max }={({{\rm{I}}}_{0}{{\rm{e}}}^{-\frac{4\pi {k}_{\max }L}{\lambda }})}^{2},{P}_{\min }={({{\rm{I}}}_{0}{{\rm{e}}}^{-\frac{4\pi {k}_{\min }L}{\lambda }})}^{2},$$

where *k*_max_ and *k*_min_ is the maximum and minimum imaginary part of the effective mode refractive index, respectively.

#### Normalized effective mode area

The normalized effective mode area is defined by (*A*_*eff*_ */A*_*0*_), where *A*_*0*_ is the diffraction-limited mode area and defined as *λ* ^*2*^ */4*, and the effective mode area *A*_*eff*_ is defined as the ratio of the total mode energy and the peak energy density which is given by

10 $${A}_{eff}=\frac{{W}_{m}}{\max \{W(r)\}}=\frac{1}{{\rm{\max }}\{W(r)\}}\int {\int }_{-\infty }^{+\infty }W(r){{\rm{d}}}^{2}r,$$

where *W*_m_ and *W*(r) are the electromagnetic energy and energy density, respectively (per unit length along the direction of propagation).

#### Other parameters

Here we briefly evaluate the modulation speed and the energy consumption of our proposed modulators. Since the two modulators have the same basic structure, their modulation speed and power consumption are approximately equal. The modulation speed which can be described by 3 dB bandwidth (*f*_3dB_) is calculated by

11 $${f}_{3dB}=\frac{1}{2\pi RC},$$

where *R* (~600 Ω) and *C* (~0.2 pF) are the total resistance and total capacitance of modulators, respectively. After considering the contact resistance and quantum capacitance of monolayer graphene^32, 33^, the modulation speed of the proposed modulators is ~1.3 GHz. And the energy consumption of our modulators are calculated by

12 $$E=\frac{1}{4}C{V}_{pp}^{2}$$

where *V*_*pp*_ ≈ 8 V is the peak-to-peak voltage here. 1/4 comes from the fact that in an NRZ signaling scheme, and for a random bit sequence, one complete charge/discharge cycle occurs on average once every four bits^32^. Thus the energy consumption of our modulators is at the level of 1 pJ/bit.
